# Proceedings of the Iowa State Dental Society

**Published:** 1865-10

**Authors:** 


### Proceedings of Societies.

### PROCEEDINGS OF THE IOWA STATE DENTAL SOCIETY.

**Held at Dubuque, Iowa, July 19th and 20th, 1865.**

(*Continued.*)

The Secretary read an essay forwarded by Dr. Tullos, on

### DECIDUOUS TEETH—THEIR IMPORTANCE—THEIR PRESERVATION.

Dr. Hardeman. — This is an important subject, and deserves our serious consideration. I have seen a great many deciduous teeth, and extracted a great many; many that I would not extract, if I had it to do over again. Yet I think one of the phrases in the essay is a little too sweeping; I

could not concur in the advice to extract *no* deciduous teeth. We do find cases where these need extraction, before the permanent teeth have made their appearance. We know that necrosis does occur, from want of cleanliness, from lack of proper care, from caries, &c. And when teeth become necrosed, a great change takes place in their relation to the vitalized parts surrounding them. In a healthy condition the root of the deciduous tooth is got rid of by absorption, and without any meddling with by operators. But when the tooth becomes necrosed, the process of absorption is arrested. Then, instead of a physiological action, a pathological condition exists. The tooth becomes to all intents and purposes a foreign body, and is productive of as much injury, with the exception of the injury that might be done by placing it there. The permanent tooth comes, as nature designed, but the way has not been prepared for it ; there is an obstacle in the way. We find the necrosed root crowded one side, or shoved forward, often penetrating the gum, sometimes to the abrasion of the lip. In such cases, I am decidedly in favor of the removal of the tooth.

Dr. Clarke—Asked for an expression from some of those present, as to the propriety of plugging the nerve-cavity of a deciduous tooth.

Dr. Chase.—I told the President not to call on me, for I could not talk ; but as Dr. Clark has asked a question in regard to plugging deciduous teeth, I will say that I am in the constant habit of doing it. When a child is brought to me with an aching tooth, if I think I could save that tooth, supposing it was a deciduous tooth, I treat it just as I would a permanent tooth. I have had just as good success in plugging and saving deciduous teeth as permanent teeth. In fact, the deciduous teeth, when thus treated, are *not so liable* to give trouble, as permanent teeth are ; they are very little liable to ulceration. Children's deciduous teeth bear treatment exceedingly well; far better than I formerly supposed.

Dr. Clarke.—If the root of a tooth is plugged with gold, how are you to get rid of the root by absorption, while the gold is there? I acknowledge I have treated teeth that way.

Dr. Chase.—I would say that I never attempt to fill the *roots* of deciduous teeth. By the pulp-cavity I mean the pulp-cavity, and not the canals of the roots. The pulp I destroy with arsenious paste, or some preparation of that kind.

Dr. Myers.—In regard to filling children's teeth, I never had a great deal of experience, for one reason; I find it a very difficult matter to control children, in such a manner as to fill their teeth satisfactorily. As we have no control of them, it is left with the parents; and very often the parents have no control of them when they come to our office. I have usually filled teeth for children with good success, but sometimes with not so good success.

In regard to extracting the deciduous teeth, I always avoid it when possible.

Dr. Peebles.—I dislike to respond to your call, for I think it is taking a privilege from the actual members for honorary members to occupy so much of the time. If my experience and observation and practice in regard to this matter will be of any benefit, however, I am perfectly willing to give them. I formerly extracted deciduous teeth, when they were aching; I seldom do it now, unless they are necrosed, and then, as Dr. Hardeman justly remarked, because the process of absorption cannot go on in the natural way. The blood-vessels being destroyed by the destruction of the periostal covering, absorption cannot go on, and the dead tooth remains, as a foreign substance, in the gum; and it is better to get it out of the way than to leave it there, a source of constant irritation. My observation and experience has taught me that the treatment of deciduous teeth with arsenious acid is equally as successful as that of permanent teeth.

The question has been asked, if we fill the fangs of deciduous teeth with gold, what would be the result? We all know the blood-vessels cannot take up gold in its crude form. But I will tell you what I have myself seen. I saw a tooth that Dr. Spalding, of St. Louis, had filled, a superior molar tooth; it was extracted afterwards, and Dr. Mc——— showed it to me; the fangs had absorbed away, leaving the gold sticking out of one fang at least two lines, and of the other full three lines; there stood the little points of gold foil; not gold wire, but gold foil.

I do not fill very far below the pulp-cavity. I introduce a little cotton, saturated with creosote, and push it gently into the canal; and then I prefer Hill's stopping. First, because it is a non-conducting agent; second, it is easy of application, as it yields under a warm instrument. As a common thing, I fill with the ordinary amalgam. Where the little patient is disposed to hold still, and I think I can make a solid gold filling, I put gold in. It is hard to keep the mouth dry long enough to pack gold well; the mouth is smaller, and to keep napkins in is hard work.

Dr. Porter.—I have been in the habit of filling deciduous teeth, more or less, for twelve years. It depends on the age of the patient presented whether I fill them or not. Little ones of the age of three or four years, their teeth I fill, of course, to preserve the arch from contracting, and leave plenty of space for the permanent teeth. When the nerves are exposed, I use oxide of cobalt, and creosote, nothing else. I let it remain an hour and a half, or two hours; the younger the patient, the less time it takes to destroy the nerve. I remove that and the next day remove the pulp, and make another application, generally of tannin and creosote, to stop the flow of blood or lymph. In the course of the fourth day from the time I remove the pulp, I fill the tooth with pure gutta-percha, or Hill's stopping. I fill that to the bulb of the nerve; above that with tin foil, or gold foil, at the option of the parent or guardian. I have no trouble afterwards, in healthy cases. As I before remarked, I always take the age of the patient into consideration. With a patient six years old, I would of course pursue a very different mode of treatment from that I have described. I should expect the permanent teeth to be sufficiently near, so that the deciduous teeth could be extracted at once; the instrument will show. I always extract, then, without the use of the lancet.

Dr. Chase.—In case of the canines—would you extract *them* at six years old? It may be seven or eight years afterwards before the permanent ones will come; it may be twenty or thirty. Hardly so long as that, though, in this western country; it is an excellent country for cutting eye-teeth!

Dr. Taft.—This subject is of too much importance to be properly treated and clearly illustrated in a few minutes.—There is not a subject of more importance in the whole range of our profession. It is a matter that most deeply concerns every one of you, if you have children, or any interest in anybody else's children; and if you have not I pity you.—It is estimated that one-sixth of all the children that die, die from diseases consequent upon difficult dentition. That is, on an average, one child out of every family in the land.—Many of those who live and "worry through childhood" sustain injuries from which they never recover; they never attain the condition constitutionally they would have attained had there been no difficulty in this process. The permanent teeth may be injured by diseases of the temporary teeth. This is very different, I know, from the common opinion of people. The most of people think, if their children can but worry through with the eruption of the temporary teeth, and then the shedding of them, all is well. But all is *not* well. The diseased condition of the temporary teeth have inflicted a lasting injury on the permanent teeth, so as to render them vastly inferior to what they would have been under other circumstances.

Another thing:—I regard dentition as a normal process; as one about which there need be no more difficulty or pain than in the expansion of the cranium, or the development or growth of any other part of the body. Then why so much difficulty here? the question will arise. For many reasons. This is a peculiar process; one about which very little is known; in fact, one about which the people generally know nothing. The fact is, we are reared, and our children are reared, in an imperfect and unnatural way; and not only the teeth, but the entire organism, suffers. The organization may be originally imperfect, or the material necessary to build up a perfect structure may be wanting — the food, or the manner of administering the food may be wrong. Add to this the local irritation caused by the eruption of the teeth, and there is enough to account for difficult dentition. Parents and everybody else interested should understand these things thoroughly, so that they might direct the whole matter, and bring it to a proper result. We should pursue that means which will keep them in the best possible condition. That comprehends the whole of it. The nourishment should be of the proper kind; that which will not only sustain it, but build it up. It should be given in such quantity, and in such manner, as will supply all the elements that go to make up its organization. It devolves upon you, the members of the profession, to see that the people do not remain in ignorance on this matter. So nourish the child, so guard its health, that there will be no interruption in the developing process anywhere. Treat a child as most children are treated — furnish it improper food, prepared in an improper manner, given at improper times, and in improper quantities—and no wonder the system becomes so morbidly sensitive that every irritating influence creates an inordinate disturbance. Avoid these things, for the sake of the teeth, if no other organ of the body. The question is not, what will you do with them after they are decayed, dead—foreign substances like a thorn in the flesh? The question is, how prevent them from attaining that condition? Give your attention to the matter long before; secure a better state of health, and so secure a better state of teeth. See that the secretions of the mouth, and all the secretions, are of the proper description. See that the teeth are kept clean. Many children, at even two or three years of age, have teeth so dirty, so covered with one filthy material and another, that you could hardly tell that they were teeth if you did not find them in the mouth. A great many eat food that does not give the teeth proper exercise; food so soft that it requires no mastication, no effort of the teeth. See that the teeth are used for the purpose for which they were intended. Prevent them from decaying.—Watch the temporary teeth as closely as you would the permanent teeth; if decay commences anywhere, have it arrested immediately. If a little spot makes its appearance, cut it away, and polish the tooth perfectly smooth and solid. If there is a cavity, fill it, either with gold, or tin-foil; or with Hill's stopping, if it is to be used for but a little while.

I never destroy the pulp of a deciduous tooth, if I can avoid it. If the pulp is exposed, I apply some sedative, to allay the pain, and fill with Hill's stopping. If you find it has died, cleanse it out, and when the inflammation has subsided, fill the roots with cotton and creosote. This is just as good as if you filled them with gold. But suppose the pulp has been dead, and it appears that an abscess is about to be formed. Clean it out. For facilitating that, use sulphuric ether and alum. When there is an inflammatory condition, cleanse out, and fill up with alum triturated in ether, laying a little pledget on the gums each side. If an abscess has formed, and you can control it so there is only a slight discharge, I should prefer to let the tooth remain, rather than to remove it. It is a terrible mistake to snatch out every tooth where there is an abscess. You are liable to destroy the germ of the permanent one.

Another point. The position of some gentlemen here seems to be, if I understand them correctly, that when the pulp of a tooth dies, it is then wholly necrosed. I cannot subscribe to that doctrine. The tooth is wholly necrosed when the periosteum is completely destroyed, and not till then.

So far as it retains its external periostal covering, it is alive. It is a beautiful provision of nature, that a tooth may die inside, and yet not die outside. So long as there is periosteum enough left to retain a tooth in its position, make an effort to save it. If the child suffers on, and on, and on, a number of days, without prospect of relief from any remedies we may apply, I might remove it, the tooth. But I do not remember when I have been brought to such a point.

Of course, when the periostal connection is entirely destroyed, absorption would not take place. The menstruum that disolves the root of the tooth is liberated from the blood; it eats off to the neck of the tooth, and the crown falls away.

Dr. Kulp.—I can add nothing whatever to what has already been said. I coincide entirely with the sentiments expressed by Dr. Taft. I can let my conscience rest easy on this subject; I believe I never prematurely extracted a deciduous tooth. I am in the habit of amputating the roots of teeth, when they cut through the gum and cause abrasion of the lip; I trim them down, I prescribe something to reduce inflammation, and *keep* it reduced till the time comes for the new teeth to make their appearance.

I have had a family of six or eight persons under my charge for three or four years. In three of the members of the family, all their front teeth are in the condition I have mentioned: the pulps died, and the roots made their appearance outside, away up, under the lip. One member of the family had three teeth extracted on account of it. The mouth of that member presents a dreadful case of irregularity. With the other members of the family I have pursued the course I have described. I have trimmed down the roots, sometimes every month, sometimes every two or three weeks—in short, whenever they have become a source of irritation. Two of the members of the family have lost those front teeth, and they are replaced by a new and perfect set—as symmetrical a set of teeth as you ever saw. My experience in these cases has taught me that it is better to suffer for the time being, than to relieve the suffering at once, and afterwards go through life with such a set of teeth as one of that family has to-day.

I make it a part of my professional duty to instruct parents and guardians within the range of my practice in the care of their teeth. In many families I can perceive quite a change in the condition of the teeth, between the older and the younger members; whether it is owing to my instruction, leading them to take better care of their teeth, I cannot say; but I am vain enough to think so. Yet I see many cases of irregularity, owing to the shameful practice of extracting deciduous teeth. If a child of mine were to be mangled in that way, I should prosecute the dentist for mal-practice.—I hope the time will come when public intelligence shall be such that they will "put us through" if we perform as some do. I hope to see some good from the action of the National Convention. It has been proposed that a committee be appointed to confer upon the subject of having something about deciduous teeth, and how to take care of them, in our common school books, so that while children are learning to spell and read they will learn something about their teeth. I am surprised at the heedlessness and ignorance of parents, and sometimes dentists, in regard to this matter. I mean—tooth carpenters. We can do much good, not only by talking in our offices, but by once in a while contributing an article to our daily papers. I have been in the habit of writing a series of letters upon the care of the teeth, and similar subjects; and I am satisfied that they have been of some benefit. If we can do good to a single family, let us do it.

Dr. Taft.—A few days since a child was brought to me, a little over three years old. The roots of three inferior incisors had penetrated through the gums, and were producing irritation. I took a pair of fine cutting forceps, and nipped off the points, laying a little pledget of cotton upon them. In a few months or weeks, those roots will probably protrude a little more, and again cause irritation, the same as before; and then I shall nip them off again. A dentist (?) told me to pull them! I told him I would sooner have the child's nose cut off! As often as once a week I am called upon to trim down these little roots. I make some simple application, and the irritation abates. If such teeth are taken out, there will almost certainly be irregularity of the permanent teeth.

Dr. Hardeman.—One question: — Whether the necrosed tooth remaining at the approach of the permanent tooth, will not do it more injury than the removal? Whether the irritation, the excitement, will not incite growth and premature advance? And whether the presence of the necrosed root will not throw the permanent tooth posterior to the proper arch where it should come?

Dr. Taft. — The irritation is mostly, or entirely on the surface. It is caused by the point of the root wearing against the lip. We cut it off to relieve that irritation. If the irritation did extend down to the permanent tooth, it would do no injury:— not a tithe of the injury, at least, that it would to take it away, and let the tissues grow up solid and firm over it.

Dr. Peebles.—Do you mean to say that a cicatrix formed is more difficult to be absorbed than a normal tissue?

Dr. Taft.—I did not say so, nor mean to be so understood. When you take out a temporary tooth, the socket it occupied closes up. For the permanent tooth, on its way to the surface, to pierce that secondary tissue, *is* more difficult, than if the socket were occupied by the temporary tooth, whose roots are absorbed before it, and the way *left clear.*

Dr. Hardeman.—I do not believe, and I presume there are not many who do, that the soft parts will present a great deal of resistance to the permanent tooth. Invariably, when the necrosed foreign body is removed, a healthy, good-colored tissue is left; and when the permanent teeth come early or late, they easily get through.

Dr. Kulp. — My objection to the removal of deciduous teeth is not so much from fear of trouble in the eruption of the permanent teeth, as from the fact that by extraction you arrest the process of nature in the formation of the dental arch, so that you have not *room* in the jaw for the permanent teeth. I do not anticipate any great deal of trouble in cutting the permanent teeth, but if the temporary teeth have been extracted long before, the absorption which follows must contract the dental arch so there is not sufficient space for the permanent set, and they must crowd upon each other, and great irregularity be produced.

Dr. Taft.—It is certain that the eruption of the permanent teeth is retarded by the premature removal of the temporary teeth. I have seen many examples — had a case but a few days ago. A child had, by accident, lost the left superior central incisor, two or three years before it should have been shed. The right superior central incisor remained its proper time, and being shed in the ordinary way, was replaced by the permanent tooth. But the left superior central incisor is retarded very much, it is not yet through the gum, but will probably make its appearance after a while.—Why does not that tooth come through, when the permanent teeth are through all around it? I presume all of you have seen cases of the same sort.

Dr. Chase. — Yes, sir — I had a case only last week. It was of a child about twelve years old. One of the central incisors was knocked out at four or five years of age, and the permanent tooth has never shown itself; the place where it ought to be is vacant to the present time. Now, an inquiry in regard to the difficulty of eruption, or the retardation of the permanent teeth by the too early extraction of the temporary ones; Is it not probably, generally the case, where temporary teeth are extracted three or four years before new ones are expected, that the sockets become filled up with bone? I do not suppose it would become filled up with bone, if the teeth below were developing, even slowly. I do not suppose nature would throw an obstacle of that sort in her own way. If she did, it would be no more difficult for her to remove *that* than the soft tissues, when the teeth are taken away too soon.

Dr. Chase read an Essay on "Anæsthetics."

The President.—This is a subject of the most vital importance we have been called upon to discuss. I hope we will be careful how we commit ourselves, individually and as a society.

Dr. Branch.—A great many years ago I used chloroform, in combination with ether. I never had any accident happen under my hands. I always used a great deal of caution, and so avoided it. During the last seven years, in the little practice I have had, I have discarded the use of anæsthetics almost entirely. My practice has been mostly country practice—which is quite different from city practice. There is a different class of patients; their ability to endure pain is quite different. I used to be afraid of chloroform; but I confess my fears have been dissipated a great deal by what I have seen in the army — in the hospitals. I have seen men for hours under the influence of chloroform. It is given without any previous preparation or examination of the patient; a man is sent ahead of the surgeons to administer it, so that the latter can commence their work as soon as they reach their patient. When they have finished operating upon him, they leave him lying on the table snoring, and let him come to when he gets ready, while they pass on to another patient. There is one difficulty accompanying its use, which has discouraged me a great deal. When a patient is brought completely under the influence of chloroform, his jaws are set, oftentimes, like a vice. I have many a time found it extremely difficult to get the forceps between the teeth. The result of my experience leads me to be discouraged in its use more from the inconvenience attending it, than from the fear of any danger to the patient. I believe where it is administered with a proper admixture of atmospheric air there is no danger in one case in a hundred; yet, the hundredth time, the patient might die. But the possibility that one man in a hundred might die under its influence, ought to make us hesitate. It has been a familiar sight to me to see men butchered, and torn to pieces, and cut to pieces, yet I have not ceased to value human life. My experience as to accidents in the use of chloroform has not been extensive, because I have been so fortunate. I have always made it a rule, rather to err on the side of caution than on the other side. During my practice I found many who were ready to use chloroform as an inducement to secure patients, and willing to take the responsibility of the consequence of its indiscriminate use; and I was willing they should. I had a great deal rather lose a patron than kill a patient. I urged them to rally all their strength to endure the temporary suffering, rather than run the risk attending the use of these agents.

Dr. Porter. — I have had seventeen years' experience in the use of anæsthetic agents. I have used what is commonly denominated lethe—composed of equal parts of Squibb's chloroform and the best ether, equal parts, by weight. In the administration of that agent, I ask all the questions necessary in regard to the constitution, whether there are any hereditary diseases in the family, &c. Where the constitution is good, and the temperament good—I prefer the bilious nervous temperament — I ask few questions. With a frail, delicate person, of nervous temperament, I ask more questions and examine closer. I examine the pulse from one to two minutes before beginning to talk about the subject; for conversation about it will make the patient nervous and agitated, and so change the movement of the pulse. I can give chloroform, I think, in any case, when it is mixed in that way. I have given it where the patients said to me, "my physicians have told me *never* to take chloroform, under any circumstances whatever." I had one case of a lady whose New York City physician had given her this special caution. Her mouth was filled with old snags, that had been there a number of years. She was of the nervous sanguine temperament, very excitable, very easily hurt, and when hurt at all, badly hurt. She knew that her teeth were a great injury to her health, but she could not endure to have them extracted without chloroform, and she had been particularly warned never to take it. I examined her pulse and was satisfied I could give her chloroform without danger, and urged her to make the trial. I began with my chloroform away out here—[at arm's length.] That is the way I do in such cases. It tends to do away with the influence of the imagination. They find they do not feel anything unusual for some time, and their imagination soon begins to silence itself. Moving it in a circle, I draw it, constantly draw it, but almost imperceptibly, closer and closer. I have then inhaled it *through the mouth*, and then when they become unconscious the mouth is still open. For this lady I extracted seven or eight very bad teeth and snags. I concluded all that were in the upper jaw. Those below I deferred till another sitting. She came out as nicely as anybody you ever saw. Next day she took the chloroform without fear.

I had another case during the winter, a very peculiar case. I could not bring the chloroform within less than six inches of her face. It appeared to rack her terribly; I was afraid of spasms. As chloroform is generally used, I presume she would have died of convulsions. I administered it to her twice, with the most satisfactory and beneficial effects.

Dr. Clarke.—I have been in the habit of using ether and chloroform from the time it commenced to be used by the profession. I extracted about eleven hundred teeth with ether, before chloroform was used at all; and about two years after that, I commenced the use of chloroform and ether in combination. I continued its use with great success till a few years since, when I had a very severe hemorrhage from the lungs, and nearly discontinued its use. I do not know that I received any injury, but I feared I should receive injury. Some years since, physicians used to bring their patients to me, to have chloroform administered, and teeth extracted. In two such cases, the patients were subject to hemorrhage from the lungs. I protested against the use of chloroform under such circumstances. The physician insisted, said it was safe, and himself administered the chloroform. No injury resulted in either case.

I invited Dr. Staples to come here to-day, and was in hopes he would come, and give us his experience in the army; but I am sorry to see he has not come. I believe dentists generally have a larger experience in the use of anæsthetic agents than physicians; but Dr. Staples, having been Division Surgeon and Medical Director in the army, has had peculiar opportunities for observing their effects. He always had a four or six horse load of chloroform accompanying him. I was much interested in conversing with him, and learned some facts of considerable importance.

I find all these army surgeons in favor of Squibb's chloroform. They think we should not use ether with it. Dr. Staples insists upon one thing. He says that when soldiers have gone into battle with loaded stomachs, having just eaten, and are wounded and brought before him — in such cases chloroform acts more unfavorably than under any other circumstances. When soldiers went into battle hungry, chloroform worked admirably. Under such circumstances, he says, he has never seen a bad result from its use. And all of these surgeons place great stress upon the importance of having the stomach empty when about to administer chloroform.

There is another thing that Dr. Chase referred to in his essay.

Some of these surgeons say it is better for the patient to take a drink of liquor, some twenty or thirty minutes before using the chloroform. They say that when the patient is brought in exhausted, it is better to give them a little ardent spirits; the effect of the chloroform is far pleasanter than without it. I should like more light upon this subject.

Dr. Porter. — I always require the preparation of my subjects the day before. If I can have such preparation before-hand, my choice of time is at ten o'clock, the patient having taken a slight breakfast, or none at all. If I cannot have such preparation, I usually select from eleven to twelve. I find that one drink of liquor makes a patient as nervous again as he otherwise would be — yes, three or four times as nervous.

I had one peculiar case, that might interest some of you. It was that of a lady from one of our best families; her husband held a state office. She sent for me ten different times. I always set my own hour—about five or half past five in the afternoon—for I knew just what sort of a time I was going to have. I would commence by looking at the tooth; I might touch the gum and I might not. I would have my forceps carelessly covered with my handkerchief calculating to snatch the tooth out suddenly, without her observing the instrument. "Well," she would say, "I am all ready, now!" I would place my hand near her mouth, and she would instantly grab hold of it. This was repeated from twenty to thirty times. She would not take chloroform under any circumstances.—Finally, I hit upon the plan of getting her intoxicated. I sent for some Irish whisky. Said I to her, "you commence at three o'clock, and take this whisky." Well, at five o'clock I went up there. I did not take any instrument with me; I knew she was not "mellow" yet. Said I, "when you have taken all this whisky, I will go after my instrument; and I shan't go before." She took the whisky, and I went and brought my instrument. When I got back she was just right; had no care, nor fear. I had the servant girl hold her hands quiet, while I extracted fifteen teeth, and she sat and laughed as I flung them on to the floor.

Since that time I have felt safe in recommending Irish whisky as an anæsthetic.

Dr. Chase.—In regard to the closing of the jaws, under the influence of chloroform: — the remedy is, give *more* chloroform. If the jaws become set, continue the chloroform, and the muscles will relax. Or, let them come to, somewhat, and place a large cork between the teeth.

In regard to the loss of life, Dr. Branch thought that if the chances of a fatal result from the use of chloroform were one in a hundred, it devolved upon us to be very cautious.—From careful estimates that have been made, the proportion of deaths from chloroform is not one in one hundred thousand, of those who use it.

In regard to the use of alcoholic drinks before administering chloroform my experience differs from that of others who have spoken. I have never been in the habit of giving it for the purpose of preparing the patient for the administration of chloroform, but when men have taken it, it has always given trouble, I always specially forbid my patients taking any alcoholic drinks, when talking about coming to have chloroform or ether administered.

Dr. Peebles.—About the year 1845 or '6, I commenced using anæsthetics. I bought the right to use the "lethe" in my office. I used that awhile, when the announcement of the discovery of chloroform came out. I tried hard to get some chloroform, but failed. The "Journal of American Dental Science" published a recipe for making chloroform. The first chloroform I ever used, I manufactured myself, in accordance with that recipe. I distilled it so rapidly as not to separate the water of distillation entirely; I let my patients inhale it from the mouth of a bottle, and produced very fine results. I afterwards went to Boston — just after Dr. Meredith had lost a patient from the use of chloroform. The price of the article was pretty well down, just then, and I laid in a good supply. I was not afraid of it, and used it with perfect fearlessness. I gave it in every case when the patient asked for it, or where I thought it best to give it.—One of my patients, a lady, was about to have a set of artificial teeth made, and I was preparing her mouth therefor.—She was a woman that had peculiar notions of her own, and one of her notions was, to have her teeth taken out at three different times — one-third at each time. They were to be taken on three successive days, at eleven o'clock in the forenoon, and she had selected just what particular teeth she wished to have taken out each time. The first day at eleven o'clock I came and took out one-third of them; the next day at eleven I went again and extracted another third; and she felt so well she thought she would like to have the rest of them taken out immediately after dinner. At one o'clock I went again, and found her still at dinner. I took my seat in the parlor, and in a few minutes she came in, in high spirits. She took her seat, and I gave her two or three inspirations of chloroform, when she *ceased to breathe*—and so did I! I did not breathe till she did; and I am not certain whether I should ever have breathed again if she had not.—Beside us sat her husband, utterly unconscious of any danger. I was perfectly astounded, and held on to the lady, utterly incapable of motion or speech. After a long time — half a minute, I presume, but half an hour it seemed to me — she gave a heard breath; and so did I. I suppose that my looks showed to the husband that "something was up." He and I laid her on the bed, and she did not go out calling that afternoon—nor did I! She was a very sick woman for quite a while. I did not give another dose of chloroform, or anæsthetic of any kind, for twelve months. I reasoned with myself thus: — if I can give it twice to the same patient, with such happy results, within two hours afterwards with almost fatal results, how am I to know when I am going to kill somebody with it, without a moment's warning? Then Simpson's paper appeared, stating the evil effects of giving chloroform upon a full stomach; and I instantly knew what was the matter. After that I began to give chloroform again, but never so freely as before. I never have given chloroform since except on an empty stomach, but once or twice, when the patient cheated me. One patient cheated me into giving him chloroform on a full stomach; he told me he had not eaten any breakfast; on the strength of that assertion I administered the chloroform; whereupon he proved himself to have lied, by throwing up about two pounds of beef steak!

With regard to the administration of chloroform to patients whose lungs are affected, I must confess I had very great fears, at the commencement of my experience. I recollect I was once applied to by a physician to administer chloroform to his wife, who was very low with consumption, in order to extract some troublesome teeth; I objected on account of the condition of her lungs; he insisted; said he would take all the responsibility; if I desired it, he would give me a written statement clearing me of all responsibility, and in case of evil results, I might publish it in the papers. I finally administered the chloroform and took out the aching teeth. It was done at her house; she had not been able to sit proped up in her bed for six weeks, she was so far gone with consumption. The next day I called to see the lady; she was sitting proped up in bed, feeling better than for a long time before; six weeks afterwards she rode out with her husband; she finally recovered entirely, her lungs healed up, or what there was left of them, expectoration ceased, and she lived for seven years, and finally died of a different disease. When I went to St. Louis; I chanced to fall in with Dr. Hunter, of New York City, famous for his treatment of pulmonary diseases. I mentioned this case to him. Said he, "In my treatment of lung diseases, the vehicle of my remedies is chloroform; and I do not know," he added, "but it does as much good without the medicine as with it." Since then I have ceased to be fearful of the effects of chloroform upon the lungs; on the contrary, all my exerience has shown that it is as useful to heal the lungs, as soap and water to cleanse the skin. But in case of cardiac diseases, organic disease of the heart, I am afraid to give it.

As I grow older, I confess I become less and less sanguine about these anæsthetic agents. I used the galvanic battery for awhile, threw it aside, took it up again, and do use it now, occasionally. I do not use ether at all. Chloroform never, if I can persuade the patient to have the teeth extracted without; and I find that care, kindness, and firmness, act very well without any anæsthetics. In St. Louis, we are particularly favored in having some very good Bourbon whisky. Sometimes giving a little of that to the patient has a wonderful influence. It makes them more brave, less fearful of danger.

One of the gentlemen, Dr. Branch, I think, spoke of the people of the country being more robust, having better *physiques*, being able to bear pain better, than those of the city. My experience does not tally with that. I practiced for about fourteen years in country towns, and have had considerable country practice since I located in St. Louis. I think my best and most heroic patients are our city ladies, who have been reared delicately, and have really delicate organizations. I find that when one of these ladies sit down to have her teeth extracted, if she has really *made up her mind*, there is little difficulty. If she comes to please her mother, or her brother, or her husband, and that only after much coaxing, and being but half-persuaded after all, she shrinks, and hesitates, and does not bear it as well as she might.

Dr. Branch.—I did not mean to say that city ladies had not an equal amount of fortitude with country ladies; I meant the *organization itself* would not suffer as much as that of a feeble person. Sometimes the person whose physical system suffers most will make no outcry nor complaint. I meant that the strong robust body of the country man or country woman would suffer less than the delicate organization of the city reared lady.

Dr. Peebles.—If we were to judge of the amount of suffering by the noise that is made, the negroes and Indians suffer most. The Indians are called a brave race; yet when you pull teeth for them, they *yell!* Negroes come next in the list; and next the low Irish. But if a lady comes to have a tooth drawn, and has made up her mind to bear it, she bears it, and that without much fuss. You remember the old rhyme—

"When she will, she will, you may depend on't;
And when she won't, she won't, and that's the end on't."

A woman is afraid of danger until she is brought face to face with it. A man has a few rotten teeth in his head, that trouble him, and some day when they ache a little more than usual, he comes into the office, with an oath, perhaps and asks you to jerk out the whole set. Place him in a chair, and by the time you have got out the first one he sputters out, "See here, old fellow, can't we wait a day or two for these others?" If it had been a woman, after you had got her courage screwed up sufficiently to have taken one tooth out, you could have taken out every tooth in her head, without her saying a word about it.

I generally give chloroform on a napkin, held near the mouth, the patient inhaling through the mouth, in order that the mouth may be open when I am ready to operate. I like the napkin starched stiff, twisted into a cone form, and hold it in front of the mouth and nose. If there is not enough chloroform to produce the proper effect, drop a little more on top of the napkin; it will soak right through.

While administering chloroform I pay no attention to the pulse—not at all; none to the general circulation; but great and marked attention to the peripheral circulation. So long as the roseate hue is kept up about the face, I know that the anæsthesia has not reached a dangerous point. I have known the pulse to drop, so as to be almost imperceptible, while still the blush was about the cheek; and I knew there was no danger.

Dr. Nichols.—I have read that in the preparation of chloroform, a peculiar poison was evolved; that by keeping the chloroform in distilled water, the poison was absorbed, and then there was no danger. Is there any truth in this statement?

Dr. Peebles.—I am no chemist, and can give no reply that would be of any importance; I am sorry more dentists are not chemists; I can only say, I have never heard of anything of the sort.

Dr. Taft.—I do not know much about anæsthetic agents experimentally. I have administered nearly every one on the list—perhaps enough to become disgusted with some of them. Chloroform is used perhaps more than any other anæsthetic agent. Yet very little is understood about its action. The state of the system in which its action would be favorable or unfavorable, the kind of cases in which it would be injurious or fatal, are mysteries to us yet. We only know that with chloroform, as with many kinds of medicine, and even of food, it operates kindly on certain constitutions, at certain times, and badly at others. Dr. Peebles has mentioned a case where to all appearance, aside from its anæsthetic qualities, chloroform appeared to be the means of restoring to health a person apparently doomed to death — showing that it possesses therapeutic powers hitherto unsuspected. In other cases, chloroform destroys life as quick as a stroke of lightning. There is an infinite variety of intermediates between these two extremes. Whether this difference in the effects of chloroform upon the system, is based upon anything tangible in the constitution, I do not know. I wish somebody would find out.

Death may be produced in several ways. Frequently death is produced by suffocation; by coagulated blood falling into the throat, and simply strangling the patient. The action of the respiratory muscles is somewhat enfeebled, and a slight pressure upon the muscles of the glottis closes it, and prevents the egress of the air from the lungs. Of course, in such cases, the course to be pursued is to clear the throat by simply throwing the head forward. I have known a number of cases of that kind, where the patient ceased to breathe, without strangling; on the mouth being opened and the tongue brought forward, the blood would pass from the mouth, and the patient begin to breathe again.

Sometimes death occurs in a different manner. The patient dies before the gum is cut, before there is any blood in the mouth at all, and dies instantly. The chloroform appears to cause a suspension of the action of the nervous centers. How it produces this effect, or why, or what is the condition of the system, to render it liable to be thus affected, we do not know. Everybody who administers chloroform does it in the dark.

I regard nitrous oxide as preferable to any other agent with which I am acquainted, for the production of anæsthesia. Its action is very different, in some respects directly the opposite from that of chloroform. While chloroform produces a diminution, a lowering, sometimes a suspension of the vital energies; nitrous oxide produces an exhilaration, or increased action of the vital powers. Chloroform tends to the deoxyganization of the blood; there is no oxygen in chloroform, to supply the place of that expended. By the inhalation of nitrous oxide, a considerable portion of oxygen is taken into the blood, and upon that depends the exhilaration of the vital forces; the blood is hyper-oxygenized. There is no case on record of death from the administration of nitrous oxide. I do not say it is impossible to produce death with it—you may administer beef-steak to a man until you kill him. With any anæsthetic the patient may suffocate from the throat filling with blood, but that is not the fault of the agent. There is no evidence that nitrous oxide produces death by suspending the operation of the nervous centers, like chloroform.

I have not used the battery much; my success with it has been but moderate; in some cases it will act well, in others not so well. Sometimes I have produced a local anæsthesia, using chloroform and tincture of aconite in equal proportions, saturating two pieces of cotton, as large as the end of the finger, placing one on each side of the tooth, and then extracting it. Whether from imagination, or from the effect of the agent, I do not know, but very frequently the patient says, "That did not hurt me at all."

On the whole, I agree with Dr. Peebles, that there is more trouble and annoyance in the use of any of these anæsthetic agents, than one in full practice can afford to be subjected to. Take a straight-forward, firm, determined course with your patients, and many a time you can dispel their fears, and they will submit everything to your judgment.

---

The foregoing is the substance of the discussions during the first day and a half of the Association. After the discussions were concluded, exhibitions were made of curiosities in the shape of unnaturally developed teeth, casts of mouths before and after difficult dental operations, dental instruments of peculiar construction, &c.

Dr. Charles exhibited a central and lateral incisor united throughout their lengths, completely, so as to look like one tooth.

Dr. ——— exhibited an undeveloped tooth found outside of the alveoli and under the gum.

Dr. Kulp exhibited casts of built up "crowns of incisors" done with gold and the mallet.

Dr. Sayles exhibited a new, simple and convenient articulator.

Dr. Chase exhibited flat crescent shaped drills, easily made and more effective than any other drills. Also, he showed the new duct compressor and tongue holder as improved by Prof. Taft.

Dr. Taft held a clinic on plugging teeth, with the use of the mallet, which was witnessed with much satisfaction by *all who could see it*. This was a *real* clinic, for Prof. Taft talked all the time, and gave his auditors as many good ideas "by word of mouth" as by his manipulations.

Several hours after the operation, the subject, Mr. Henion, a merchant of Dubuque, but formerly a dentist, expressed his great satisfaction at the manner in which the operation had been performed. He had supposed he would be hurt by the "hammering," and was surprised to find that he was not. He at first was tempted to inquire what the wedge was driven between his teeth for, but he soon found out. The wedge did away with the percussion. The use of the mallet he considered the best way of filling teeth he had ever seen yet. This tooth had been very sensitive at times. Two or three dentists had attempted to fill it; would go to work and press the filling in, but he would have to take it out again. Now it did not feel sensitive at all — not so much so as before it was filled. He should always recommend wedging, and the use of the mallet.

The afternoon of the second day was mostly devoted to business. A number of resolutions were adopted, thanking the Northern Line Packet Co., for carrying the members at half-fare rates—Mr. Russ, their host at the Julian House—the Dentists of Dubuque, for their endeavors to make the stay of their brethren while in the city agreeable—the Board of Education, for the free use of their rooms—Dr. Taft, of Cincinnati, and Dr. Peebles, of St. Louis, for their instructive remarks—a resolution in favor of the formation of local dental societies, and others of minor importance.

The following resolutions were unanimously passed, the Society voting that each of its members should endeavor to secure their publication in the local journals of the vicinity:

In Consideration of the vital importance of parental watchfulness, and early instruction as to the care and preservation of the first, or deciduous teeth, the premature loss of which leads not only to frightful irregularity, disfiguration and fatal injury of the permanent teeth, but in a very alarming proportion of cases extends even to death itself, therefore,

*Resolved*, That we declare it the sense of this meeting, that it becomes the imperative duty of parents, guardians, physicians, and others upon whom the care of children may devolve, to inform themselves thoroughly in regard to the general requirements of first dentition, and to early impress the same upon the youthful mind.

*Resolved*, That we commend the moral courage of that dentist who conscientiously refuses to inflict a lasting injury upon the mouths of the young by the premature removal of these teeth, preferring rather to adhere to the path of known duty than to yield to the blind dictation of mistaken ignorance.

Dr. Ingersoll offered the following resolution:

WHEREAS, The Iowa State Dental Society has, without a precedent, elected to membership a lady practitioner of dentistry, and

WHEREAS, It is due to her to know that the unanimous vote by which she was elected was not simply a formal vote, and

WHEREAS, It is due to the profession at large, that we make a formal declaration concerning the position we have assumed in our action, therefore,

*Resolved,* That we most cordially welcome Miss LUCY B. HOBBS, of McGregor, to our number, and to our professional pursuits, trials, aims and successes.

*Resolved,* That the profession of dentistry, involving, as it does, the vital interests of humanity, in the relief of human suffering, and the perpetuation of the comforts and enjoyments of life in civilized and refined society, has nothing in its pursuits foreign to the instincts of women, and, on the other hand, presents in almost every applicant for operations, a subject requiring a kind and benevolent consideration of he most refined womanly nature.

Brief remarks were made by several gentlemen on each of he foregoing resolutions.

The following members were appointed essayists at the next meeting: Drs. Porter, Clark, Hardeman, Severance, Branch, Sayles, and Miss Hobbs. Volunteers—Drs. Ingersoll, Chase and Kulp.

Miss Hobbs in a very brief and very neat speech, thanked the Society for all the kindness manifested towards her. She deemed the reception she had met with among them, an ample recompense for all the rebuffs and discouragements she had received during the past four years, while fighting her way into and in the profession, and expressed her determination to make her mark in the profession, so that they should never regret the step they had taken.

Dr. Peebles.—I feel I cannot go away without expressing to you how much I have learned, and how much I have been benefited. I shall go home carrying the impression of these faces upon my memory and upon my heart. I shall go away from here a better man.

My professional brethren, I feel more keenly than ever that I cannot afford—I am too poor a man to afford—to stay away from our National Association meeting, next week. I think it was worth a thousand dollars to me last year, I think I shall make it worth that to me this year. I mean in dollars and cents. I have been able to do better work, and have received better prices for it. I believe the good Lord blessed me for going there. I believe the Lord blesses us for doing our duty, and I believe that I am as much doing my duty when I go where I shall learn how to perform my duty better to my fellow men, as when I go to church and hear a sermon.

Prof. Taft has said so much in praise of St. Louis, that I do not feel as if it were my place to say anything. I will only add that we have a warm place and large hearts, and if any of you can make it in your way to call upon us you shall meet a hearty welcome. Come down.

After which the society adjourned.

